# LI-cadherin *cis*-dimerizes in the plasma membrane Ca^2+^ independently and forms highly dynamic *trans*-contacts

**DOI:** 10.1007/s00018-012-1053-y

**Published:** 2012-07-28

**Authors:** Thilo Bartolmäs, Caroline Hirschfeld-Ihlow, Sven Jonas, Michael Schaefer, Reinhard Geßner

**Affiliations:** 1 Institute for Transfusion Medicine, Charité, 13353 Berlin, Germany; 2 Center for Reproductive Medicine, 26789 Leer, Germany; 3 Rudolf-Boehm-Institute of Pharmacology and Toxicology, Leipzig University Medical School, 04103 Leipzig, Germany; 4 Laboratory for Molecular and Cellular Surgery, Department of Abdominal, Thoracic, Vascular and Transplant Surgery, Leipzig University Medical School, Liebigstr. 20, 04103 Leipzig, Germany; 5 Department of Abdominal, Thoracic, Vascular and Transplant Surgery, Leipzig University Medical School, Liebigstr. 20, 04103 Leipzig, Germany

**Keywords:** Cadherin-mediated cell–cell adhesion, Calcium ions, *Cis*-binding and *trans*-interaction, Fluorescence redistribution after photobleaching, Fluorescence resonance energy transfer

## Abstract

LI-cadherin belongs to the family of 7D-cadherins that is characterized by a low sequence similarity to classical cadherins, seven extracellular cadherin repeats (ECs), and a short cytoplasmic domain. Nevertheless, LI-cadherins mediates Ca^2+^-dependent cell–cell adhesion and induces an epitheloid cellular phenotype in non-polarized CHO cells. Whereas several studies suggest that classical cadherins *cis*-dimerize in a Ca^2+^-dependent manner and interact in *trans* by strand-swapping tryptophan 2 of EC1, little is known about the molecular interactions of LI-cadherin, which lacks tryptophan 2. We thus expressed fluorescent LI-cadherin fusion proteins in HEK293 and CHO cells, analyzed their cell–cell adhesive properties and studied their cellular distribution, *cis*-interaction, and lateral diffusion in the presence and absence of Ca^2+^. LI-cadherin highly concentrates in cell contact areas but rapidly leaves those sites upon Ca^2+^ depletion and redistributes evenly on the cell surface, indicating that it is only kept in the contact areas by *trans*-interactions. Fluorescence resonance energy transfer analysis of LI-cadherin-CFP and -YFP revealed that LI-cadherin forms *cis*-dimers that resist Ca^2+^ depletion. As determined by fluorescence redistribution after photobleaching, LI-cadherin freely diffuses in the plasma membrane as a *cis*-dimer (*D* = 0.42 ± 0.03 μm^2^/s). When trapped by *trans*-binding in cell contact areas, its diffusion coefficient decreases only threefold to *D* = 0.12 ± 0.01 μm^2^/s, revealing that, in contrast to classical and desmosomal cadherins, *trans*-contacts formed by LI-cadherin are highly dynamic.

**Electronic supplementary material:**

The online version of this article (doi:10.1007/s00018-012-1053-y) contains supplementary material, which is available to authorized users.

## Introduction

Cadherins are membrane glycoproteins acting as Ca^2+^-dependent cell–cell adhesion molecules in metazoan organisms [1, 2]. Classical and desmosomal cadherins consist of five extracellular cadherin repeats (ECs), a single transmembrane domain, and a large cytoplasmic region that binds to armadillo proteins and interacts with the cytoskeleton [3, 4]. Each EC has a length of about 110 amino acids, carries four acidic Ca^2+^-binding motifs [2, 5], and adopts a highly conserved β-barrel conformation [6, 7]. The cytoplasmic interactions with actin and intermediate filaments induce classical and desmosomal cadherins to cluster into adherens junctions and desmosomes, respectively [8–10]. Cadherin-mediated cell–cell adhesion is not a static phenomenon but a well-regulated process essential for cell division, cell migration, tissue formation, and morphogenesis [11–14].

LI-cadherin is a non-classical cadherin that was initially discovered in rat liver and intestine [15]. In human and mice, LI-cadherin is exclusively expressed in the small and large intestine but missing in the upper gastric tract and the liver [16, 17]. LI-cadherin has a low sequence similarity to classical cadherins, and consists of seven extracellular cadherin repeats, a single transmembrane domain, and a short cytoplasmic region of only about 20 amino acids [18]. Together with its kidney-specific paralogue, Ksp-cadherin, LI-cadherin forms the 7D-cadherin family [19]. Despite its lack of binding to catenins or other cytoplasmic components, LI-cadherin mediates a Ca^2+^-dependent cell–cell adhesion [20] that is comparable to that of Ksp-cadherin and classical E-cadherin [21]. Likewise, the extracellular domain of LI-cadherin exhibits a similar homotypic binding affinity (*K*_D_ = 27 μM) and single bond lifetime (τ_0_ = 1.4 s) in vitro as classical cadherins [22]. Within the intestinal epithelium, LI-cadherin is coexpressed with E-cadherin [16, 17]. However, it is excluded from adherens junctions and desmosomes, and evenly distributed in all non-specialized lateral membrane areas [15, 20].

Based on electron microscopic studies of C-terminally oligomerized E-cadherin ectodomains [23, 24] and on two crystal structures of the first two N-terminal E-cadherin repeats EC12 [24, 25], a model for the mechanism of cadherin-mediated adhesion has been proposed [24], which assumes increasing Ca^2+^ concentrations to stabilize first the cadherin ectodomains in an elongated, rod-like conformation at 50–500 μM, then to induce *cis*-dimerization at 500–1,000 μM, and finally to lead at more than 1,000 μM to *trans*-interactions of cadherins originating from opposing cell surfaces.

These functional studies were in line with the first structural analysis of a cadherin repeat, EC1 of N-cadherin [6], which revealed a partial β-strand exchange of the first N-terminal residues, including tryptophan 2 (W2), between two symmetry-related, parallel-oriented EC1 repeats. However, in a subsequent crystal structure analysis, the complete C-cadherin ectodomain was found to assume a highly curved conformation, which led to the conclusion that the Ca^2+^-independent ‘strand-dimer’ interaction involving W2 is responsible for the *trans* interaction between two cadherins emerging from the opposing surfaces of two adjacent cells [7]. These and other structural studies also revealed that clusters of Ca^2+^ ions are located in the interface between successive cadherin repeats, supporting the previous observation that Ca^2+^ binding stabilizes cadherin ectodomains in an elongated, curved conformation [23].

T-cadherin, which lacks W2 in its EC1 repeat, exhibited a different mode of interaction, termed ‘X-dimer’, that involves contacts between EC1 and EC2 [26] and was previously identified in crystal structures of E-cadherin EC12 repeat dimers [24, 25]. This interaction was also observed in crystal structures of classical cadherins lacking W2, whereas native E- and N-cadherin ectodomains exhibited the same ‘strand-dimer’ interaction as the C-cadherin ectodomain. It was thus concluded that the ‘X-dimer’ constitutes an intermediate conformation that leads in classical cadherins to the more stable ‘X-dimer’ conformation [27, 28]. This assumption was further supported by a functional study, which revealed in addition that classical cadherins most likely leave adherens junctions by first transiting from the ‘strand dimer’ into the ‘X-dimer’ interaction before they become fully separated [29].

In view of these considerations, we investigated whether non-classical LI-cadherin, which lacks tryptophan-2, forms *cis*-dimers on the cell surface and within cell–cell contacts. In addition, we analyzed the response of LI-cadherin *cis*- and *trans*-interactions as well as its cellular distribution to sudden changes in the extracellular Ca^2+^ concentration. As experimental approach, we attached cyan and yellow fluorescent protein tags to the short intracellular C terminus of LI-cadherin, analyzed the *cis*-interaction of the resulting fusion proteins in the plasma membrane by fluorescence resonance energy transfer (FRET), studied their lateral mobility by fluorescence redistribution after photobleaching (FRAP), and their response to Ca^2+^ depletion by confocal imaging, FRAP and FRET. Our analysis revealed that LI-cadherin *cis*-dimerizes constitutively and is highly mobile not only outside but also within cell–cell contacts. Upon Ca^2+^ depletion, LI-cadherin readily diffuses out of the cell contact areas and redistributes—still as a dimer—on the entire cell surface.

## Materials and methods

### Expression plasmids, cell culture and transfection

LI-cadherin cDNA [21] was adapted by PCR and ligated in frame via a Xba-I restriction site into the custom-made vectors pcDNA3-CFP or pcDNA3-YFP [30] to generate expression plasmids encoding LI-cadherin that is C-terminally tagged with cyan (CFP) or yellow (YFP) fluorescent proteins. The L221K mutation was introduced into the CFP and YFP fusion parts by PCR-mutagenesis of the expression plasmids using the primers 5′-CACATGGTCCTGAAGGAGTTCGTGACCGCC-3′ and 5′-CCCGGCGGCGGTCACGAACTCCTTCAGGAC-3′ in combination with pfuTurbo^®^ DNA polymerase and subsequent digestion of the parental plasmid DNA with Dpn I according to the QuikChange^®^ Site-Directed Mutagenesis protocol (Stratagene/Agilent Technologies, Waldbronn, Germany). All cloned constructs were confirmed by DNA sequencing on an ABI-Prism 377 sequencer (Perkin Elmer, Norwalk, CT, USA).

Human embryonic kidney (HEK293) cells (ATCC, Manassas, VA, USA) were maintained in a semi-confluent state at 37 °C and 5 % CO_2_ in minimal essential medium with Earle’s salts supplemented with 10 % fetal calf serum, 2 mM glutamine, 100 μg/ml streptomycin, and 100 U/ml penicillin. For fluorescence microscopy and confocal imaging, cells were seeded on glass coverslips. HEK293 cells were transfected with FuGENE-6 transfection reagent (Roche Applied Science, Penzberg, Germany) using 2 μg total plasmid cDNA per 35-mm dish.

Chinese hamster ovary (CHO) cells were transfected using Genejammer Transfection reagent (Agilent Technologies, München, Germany). Clones expressing LI-cadherin-YFP fusion protein (LI-YFP) or free YFP were selected in Dulbecco’s modified Eagle’s medium (DME) with 10 % fetal calf serum (FCS) and 250 μg/ml G418 for 3 weeks.

### SDS-PAGE and immunoblotting

Whole cell lysates were separated by SDS-PAGE and electroblotted onto polyvinylidene difluoride membranes (Hybond-P; Amersham Pharmacia Biotech, Freiburg, Germany). Blot membranes were incubated for at least 1 h with the following primary antibodies: rabbit anti-mLI-cadherin EC1 domain antibody (1:10,000, [22]); rabbit anti-GFP antibody (1:100, *Aequorea victoria* peptide antibody; Clontech Laboratories, CA, USA) and rabbit anti-actin antibody (1:1,000, A 2066; Sigma). After washing, the membranes were exposed for 1 h to horseradish peroxidase-conjugated, polyclonal secondary antibodies (swine anti-rabbit; Dako Cytomation, Glostrup, Denmark). Antibody complexes were visualized using the ECL plus detection kit (Amersham Pharmacia Biotech) and Biomax films (Kodak, Stuttgart, Germany).

### Hanging drop cell aggregation assay

The hanging drop cell aggregation assay was performed as described previously [21]. CHO cells stably expressing LI-YFP or non-fused YFP were trypsinized and resuspended at 10^5^ cells/ml in DME containing 10 % FCS. Droplets of 10 μl, containing about 1,000 cells, were placed on the inner side of an inverted Petri dish lid. The lid was subsequently turned back and positioned on a Petri dish filled with 10 ml PBS to avoid evaporation of the hanging cell culture droplets. After 15 min, micrographs were recorded of the hanging drops, and the number of particles (*N*_0_) was counted. After 16 h incubation at 37 °C, the number of particles was counted again. Each single cell and each cell in a coincidental cluster of 2–4 cells was defined as one particle, whereas every aggregate containing more than five cells was counted as one particle irrespective of the number of cells it contained. Individual cells and cell aggregates were summed up (*N*_t_) and cell aggregation was expressed by the aggregation index (*N*_0_ − *N*_t_)/*N*_0_ [31]. The cell aggregation index was determined in at least nine independent experiments. For each transfected cell line, three independent clones were analyzed.

### Confocal imaging and fluorescence redistribution after photobleaching

Imaging experiments were performed 36–48 h after transfection at 37 °C in a buffer containing 10 mM HEPES (pH 7.4), 128 mM NaCl, 6 mM KCl, 1 mM MgCl_2_, 2 mM CaCl_2_, 5.5 mM glucose, and 0.2 % (w/v) bovine serum albumin. In Ca^2+^ removal experiments, the buffer was exchanged by an identical buffer lacking CaCl_2_. EGTA was added at *t* = 0 to a final concentration of 2 mM. An inverted confocal laser-scanning microscope (LSM 510 Meta; Carl Zeiss) and an α-Plan-Fluar 100x/1.45 objective (Zeiss) were used for confocal imaging and FRAP analysis. YFP-tagged LI-cadherin was excited and photobleached with the 488 nm laser line of an Ar^+^ laser. Emission was recorded through a 505-nm-long pass filter. Pinholes were adjusted to yield optical sections of 0.8–1.4 μm. In a typical bleaching experiment, after 10 pre-bleach scans, a defined part of a cell–cell contact was photobleached and postbleach images were recorded until the fluorescence intensity of the bleached area had reached at least 80 % of that of neighboring unbleached areas. The first postbleach image was regarded as the initial condition and the following images were used to fit the mobility parameters [32, 33]. Means and SE of the LI-YFP diffusion coefficient were computed from at least 24 experiments of 3–6 independent transfections.

### Fluorescence resonance energy transfer

FRET experiments were performed 36–48 h after transfection at 37 °C in the same buffer as used for confocal imaging and FRAP experiments. An inverted microscope (Axiovert 100; Zeiss) with a Plan-Apochromat 63x/1.4 objective (Zeiss) was used for determining FRET efficiencies by monitoring the donor (CFP) unquenching during acceptor (YFP) photobleaching. Monochromatic light (excitation of CFP: 410 nm; of YFP: 515 nm) derived from a Xenon lamp (Polychrome IV; TILL Photonics) was passed through a dual reflectivity dichroic mirror (<460 and 500–520 nm; Chroma) for excitation. Fluorescence signals were passed through 475–505 nm (CFP) or 535–565 nm (YFP) band pass filters mounted on a motorized wheel (Lambda 10/2; Sutter Instruments), and detected by a cooled CCD camera (Imago; TILL Photonics). FRET efficiencies were determined using the previously described acceptor photobleaching protocol (JBC 2005; Philipp Voigt). The acceptor photobleaching protocol consisted of 15 prebleach cycles with 40 ms per cycle exposures at 410 nm for CFP detection and 8 ms per cycle at 515 nm for YFP detection. During the following 40 cycles, YFP was photobleached by applying an additional 1.8 s per cycle illumination at 512 nm, typically yielding about 95 % photobleaching of YFP. The relative CFP and YFP fluorescence intensities of single cells were determined and compared to those of an intramolecularly fused CFP–YFP tandem protein [34], to obtain the molar ratio between the coexpressed CFP- and YFP-tagged proteins. All measurements with excessively high concentrations of either cadherin were discarded. Only cells with acceptor-to-donor ratio exceeding 1.5 were included in the calculation of mean FRET efficiencies. FRET efficiencies *E* were calculated using the equation $E = 1 - (F_{\text{DA}} /F_{\text{D}} )$, with *F*_DA_ representing the CFP fluorescence measured before bleaching YFP and *F*_D_ representing the CFP fluorescence in absence of YFP acceptor. *F*_D_ was obtained by linear regression of the increase in CFP fluorescence with the decrease in YFP fluorescence and extrapolation to zero YFP fluorescence, i.e., complete YFP photobleach. In each experiment, data of 3–9 single cell–cell contacts with appropriate molar ratio were averaged. Means and SE were computed from 9 to 15 independent FRET experiments of 3–6 independent transfections.

## Results

### Expression of fluorescent cadherin fusion proteins

**Fig. 1:**
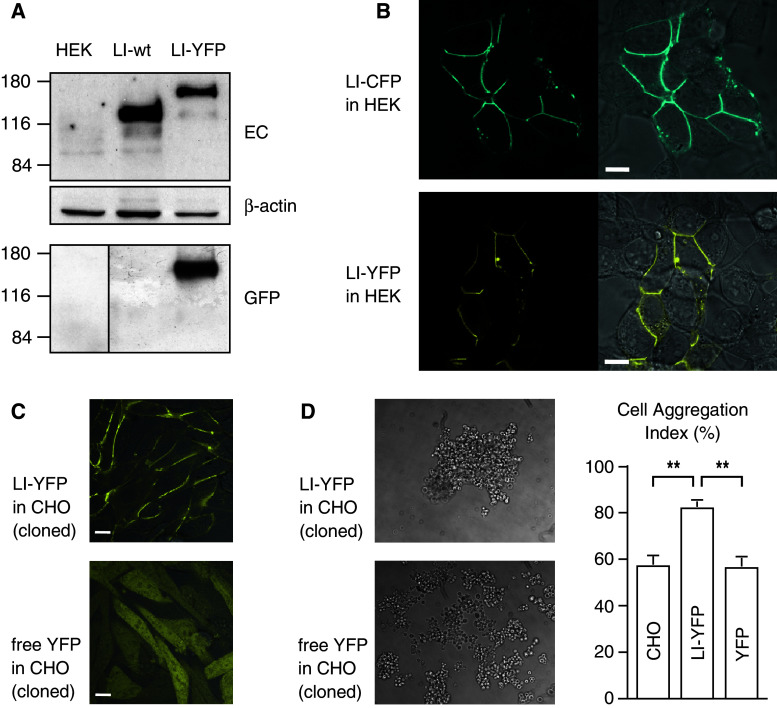
Expression of fluorescent LI-cadherin fusion proteins in HEK293 and CHO cells. **a** Western blot analysis of human embryonic kidney cells (HEK293) 48 h after transfection. Total protein (10 μg) was probed with polyclonal antibodies raised against the extracellular domain (*EC*) of LI-cadherin and with polyclonal antibodies against GFP-peptides (*GFP*). β-actin served as a loading control. **b** Confocal laser scanning microscopy of LI-cadherin CFP and YFP fusion proteins in transiently transfected HEK293 cells. Cells were imaged 48 h after transfection. *Left panels* fluorescence signal, *right panels* overlay of fluorescence signal with a Nomarski interference contrast image. *Top row* LI-CFP-transfected cells, *bottom row* LI-YFP-transfected cells. *Scale bars* 10 μm. **c** Morphology of stably transfected Chinese hamster ovary (*CHO*) cells. Overlayed confocal fluorescence and Nomarski interference contrast image of CHO cells expressing LI-YFP (*top*) and free YFP (*bottom*). LI-cadherin is efficiently targeted to the plasma membrane whereas free YFP is evenly distributed within the cytoplasm. *Scale bars* 10 μm. **d** Hanging drop adhesion assay of stably transfected and parental CHO cells. *Left* aggregation pattern of stably transfected, initially dissociated CHO cells expressing LI-YFP (*top*) or free YFP (*bottom*) after 16 h of incubation in the hanging drop. *Right* cell aggregation index (*N*_0_ − *N*_t_)/*N*_0_ of LI-YFP- and free YFP-expressing CHO cells as well as of parental CHO cells in mean ± SE; **p < 0.00001

Constructs encoding LI-cadherin with C-terminally attached cyan (LI-CFP) or yellow (LI-YFP) fluorescent protein tags were expressed in HEK293 and CHO cells. Correct expression of the fusion proteins was verified by western blotting and confocal imaging (Fig. 1a, b). The fusion proteins exhibited the expected apparent molecular mass and were detectable with antibodies directed against the first extracellular cadherin repeat of LI-cadherin as well as with antibodies against the fluorescent protein. In both cell types, the LI-cadherin fusion proteins were efficiently transported to the plasma membrane and accumulated in cell–cell contact sites (Fig. 1b, c).

### Cell–cell adhesive properties of the fusion proteins

To demonstrate the cell–cell adhesive properties of the fluorescent cadherin fusion proteins, CHO cells were stably transfected with LI-YFP or free YFP expression constructs. The cloned cells exhibited a homogenous expression level of the fluorescent proteins. Whereas free YFP was evenly distributed in the cytoplasm, LI-YFP was efficiently targeted to the plasma membrane (Fig. 1c). Cells expressing free YFP retained the spindle-shaped cellular phenotype typical of the parental CHO cells. In contrast, LI-YFP expression induced the CHO cells to adopt an epitheloid phenotype undistinguishable from CHO cells stably expressing unmodified LI-cadherin [21]. LI-YFP accumulated in cell–cell contact areas and was simultaneously depleted in membrane areas not in touch with other cells (Fig. 1c). To quantify the cell–cell adhesive properties, LI-YFP- and free YFP-expressing cell clones as well as the parental CHO cells were subjected to the previously described [21] hanging-drop cell aggregation assay (Fig. 1d). CHO cells expressing LI-YFP exhibited a cell aggregation index (*N*_0_^ ^− *N*_t_)/*N*_0_ of 0.819 ± 0.023 and formed large, irregular cell clusters. The aggregation indices of wild-type (0.570 ± 0.035) and free YFP-expressing CHO cells (0.562 ± 0.037) were significantly lower (*p* < 0.00001). These numbers correspond to those obtained with CHO cell clones expressing wild-type LI-cadherin [21]. Our results show that the cell adhesive function of LI-cadherin was not altered by attaching CFP or YFP fluorescent protein tags to its intracellular C terminus.

### LI-cadherin cis-dimerizes in living cells

**Fig. 2:**
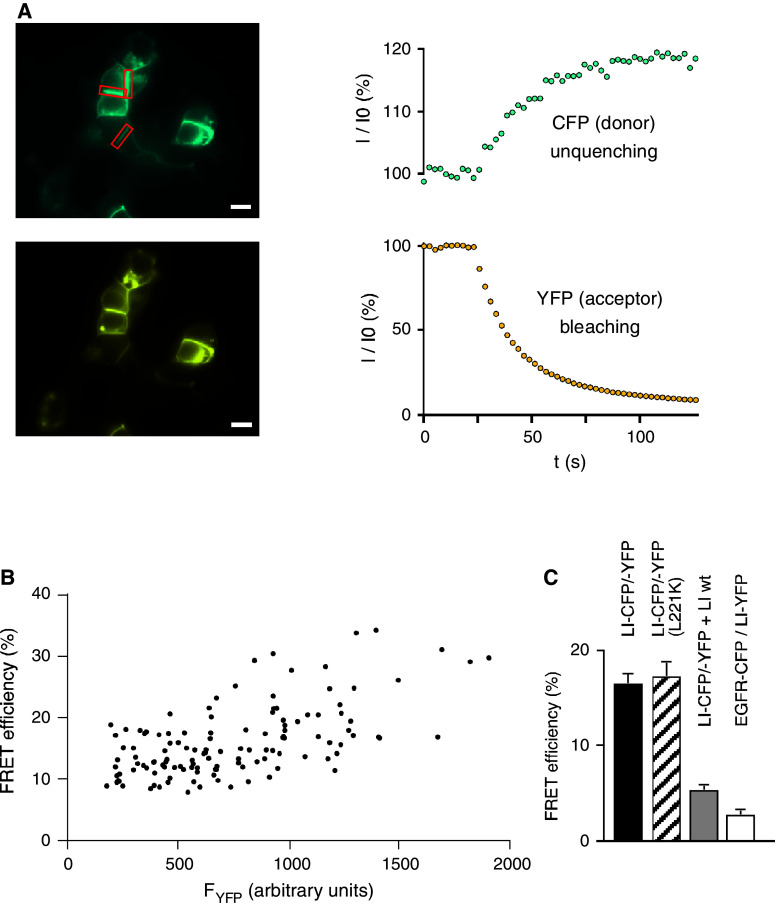
FRET analysis of fluorescent LI-cadherin-expressing HEK293 cells. **a** Acceptor bleaching experiment of double transfected cells (LI-CFP and LI-YFP). *Left side* fluorescence images in the CFP channel (*top*) and the YFP channel (*bottom*). The *red boxes* indicate cell–cell adhesion sites used for FRET analysis. *Scale bar* 10 μm. *Right side* time curve of CFP fluorescence (*top*) and YFP fluorescence (*bottom*) in a typical YFP-bleaching experiment. **b** FRET efficiencies between LI-CFP and LI-YFP over a wide range of the YFP fluorophore concentration. **c** FRET between LI-CFP and LI-YFP (LI-CFP/-YFP, *filled column*); FRET between LI-CFP and LI-YFP after introducing the L221K mutation into the CFP and YFP fusion parts (LI-CFP/-YFP (L221K), *hatched column*); FRET competition assay with LI-CFP, LI-YFP and LIwt (wild-type LI) (LI-CFP/-YFP + LIwt, *gray column*); FRET between EGF-receptor (EGFR-CFP) and LI-YFP (EGFR-CFP/LI-YFP, *white column*). FRET efficiencies as mean ± SE

After showing the correct expression, localization, and adhesive function of the fluorescent cadherins, we performed FRET experiments to probe for *cis*-dimerization. HEK293 cells were cotransfected with CFP- and YFP-fused LI-cadherin constructs and well-defined cell–cell contact sites of attached double-transfected cells were analyzed by acceptor photobleach FRET experiments (Fig. 2a). The average FRET efficiency *E* in those regions of 16.4 ± 0.9 % indicates a homotypic *cis*-dimerization of LI-cadherin (Fig. 2a).

To assess the specificity of the detected FRET efficiency, we performed the following experiments. The FRET efficiency was measured over a wide range of fluorophore concentrations showing only a minor concentration dependence of FRET efficiencies (Fig. 2b). Introducing into both fluorescent proteins a point mutation (L221K), which was previously shown to decrease the weak intrinsic interaction between CFP and YFP by two orders of magnitude without loss of quantum yield [35], did not significantly change the FRET efficiency (*E*_L221K_ = 17.1 ± 1.7 %, *p* = 0.66; Fig. 2c). In contrast, coexpression of a twofold excess of untagged LI-cadherin with LI-CFP and LI-YFP in a competition experiment [36], decreased the FRET efficiency to *E*_comp_ = 5.2 ± 0.4 %, *p* < 10^−38^ (Fig. 2c). Coexpression of LI-YFP and EGFR-CFP (epidermal growth factor receptor-CFP fusion protein), which are not known to interact, served as a negative control and yielded a FRET efficiency of only 2.7 ± 0.4 %, which is regarded as unspecific (Fig. 2c). Taken together, these results indicate that a specific *cis*-dimerization is inherent to LI-cadherin and not due to an interaction of the fluorescent proteins or an altered conformation of the fusion protein.

### Influence of Ca^2+^ on the function of LI-cadherin

**Fig. 3:**
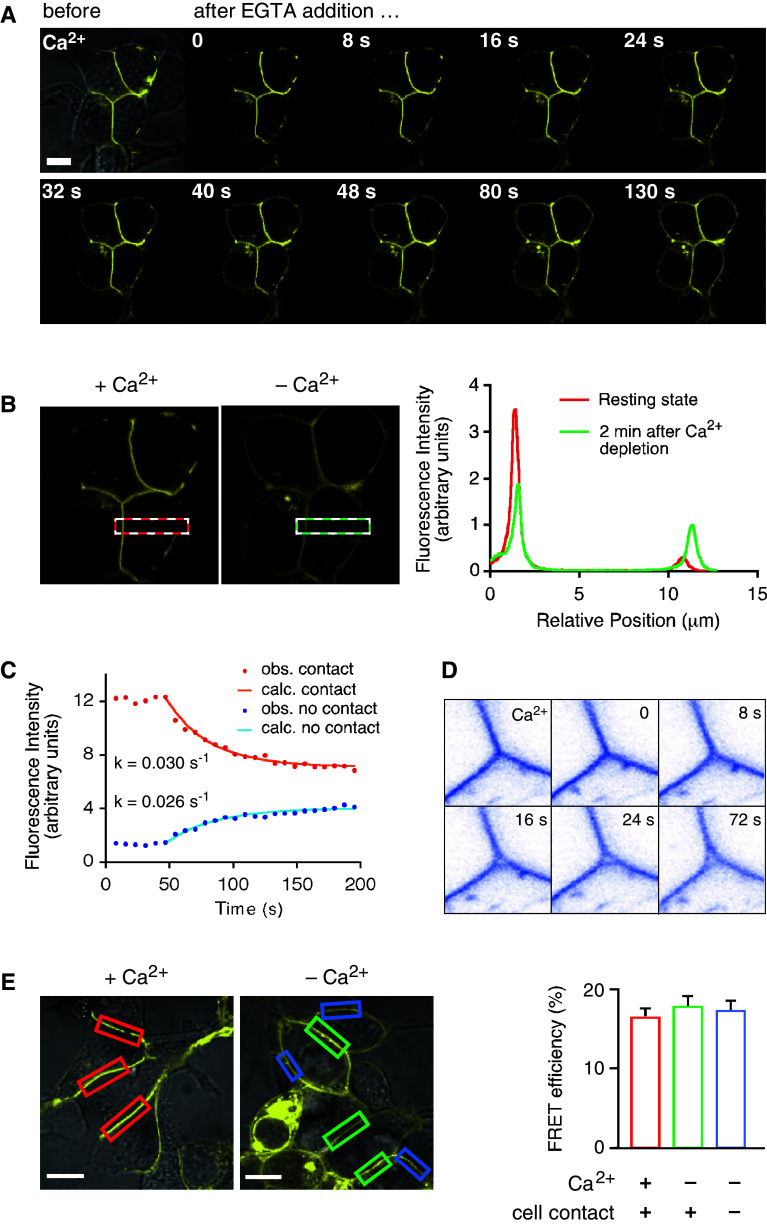
Influence of Ca^2+^ depletion on the cellular distribution and lateral association of LI-cadherin. **a** Confocal imaging of time-dependent re-distribution of LI-YFP after EGTA-induced Ca^2+^ depletion. At *t* = 0, EGTA was added to a final concentration of 2 mM. For each frame the time is indicated after addition of EGTA. *Scale bar* 10 μm. **b** Quantitative analysis of the Ca^2+^ dependent re-distribution of LI-YFP in membrane regions engaged in cell–cell contacts and those not facing other cells. The *dashed red box* in the *left micrograph* indicates the analyzed area in the presence of Ca^2+^ and the *dashed green line* in the *right micrograph* the same region of interest 2 min after Ca^2+^ depletion by EGTA addition. The fluorescence intensity was integrated along the short axis of the box and plotted along its long axis in the graph on the *right side*. **c** Time course of the cellular re-distribution of LI-YFP (*left*) and of the separation of attached membrane sheets (*right*) subsequent to Ca^2+^ depletion. The decline of fluorescence in the cell contact region following the addition of EGTA is plotted in *red* and the increase in the free membrane area in *blue*. Both curves follow a first order kinetic with similar rate constants of about 0.03 s^−1^. The data were obtained by integrating the fluorescence intensity in the respective membrane regions and plotting it over time. The time course of both graphs was approximated by least squares minimization to derive the rate constants; the calculated time curves are plotted over the observed data points in *orange* and *light blue*, respectively. In the *right panel* a tricellular junction is shown in *inverse colors*. In the presence of Ca^2+^ LI-YFP concentrates in the tricelllar junction and keeps it tightly sealed. Little changes over 20 s until EGTA is added at time *t* = 0. Just 8 s later, a small hole has formed at the center of the junction that increases in size over the next 16 s and remains basically unchanged from then on. **d** Ca^2+^-dependent FRET analysis of LI-CFP/LI-YFP-expressing HEK293 cells. *Left* fluorescence images (YFP channel only) of typical membrane regions used for FRET experiments. Regions of interest were cell–cell contacts in the presence of Ca^2+^ (marked in *red*), former cell–cell contacts after Ca^2+^ removal (marked in *green*) and membrane regions not engaged in cell contacts (marked in *blue*). *Scale bars* 10 μm. *Right* FRET efficiencies determined for LI-CFP/LI-YFP in the indicated membrane regions. Displayed are means ± SE

A hallmark of the cadherin-mediated cell–cell adhesion is its dependence on Ca^2+^ ions [1, 37]. We have recently shown by atomic force microscopy (AFM) analysis that the *trans*-interaction of LI-cadherin exhibits an extremely high cooperativity with respect to the Ca^2+^ concentration (*n*_H_ > 12). LI-cadherin thus acts as a Ca^2+^-dependent cell–cell adhesion switch that loses its adhesive properties abruptly if the Ca^2+^ concentration drops below 700 μM Ca^2+^ [22]. In view of these findings, we analyzed the effect of Ca^2+^-depletion on cellular LI-cadherin localization and interaction. Exchanging Ca^2+^-containing HEPES-buffered saline (HBS) by a Ca^2+^-free HBS had no immediate effect on LI-YFP distribution. However, the subsequent addition of the Ca^2+^ specific chelator EGTA to a final concentration of 2 mM induced LI-cadherin to rapidly leave the cell–cell contact sites and to redistribute evenly in the plasma membrane (Fig. 3a; Video 1 in Online Resource).

For one of the cell–cell contacts shown in Fig. 3a and a related non-contact region, we quantified the change in fluorescence intensity following Ca^2+^ depletion (Fig. 3b). The fluorescence intensity was projected on the long axis of the boxes shown on the left panel of Fig. 3b and plotted along this axis for the resting state (with Ca^2+^, average of first 5 time frames prior to Ca^2+^ depletion; shown in red in the right panel of Fig. 3b) and 2 min after Ca^2+^ depletion (last 5 frames; shown in green in the right panel of Fig. 3b). Initially, the integrated fluorescence intensity within the cell–cell contact (270 nm peak width) was almost nine times as high as that of the neighboring non-contact region. Within 2 min after Ca^2+^ depletion, the fluorescence intensity dropped to about 60 % within the cell–cell contact and increased almost threefold in the non-contact region, resulting in a fluorescence ratio of contact to non-contact area of about 1.8, as is expected due to the superposition of the membranes of two LI-YFP-expressing cells in the contact area (Fig. 3b, right panel). The Ca^2+^ depletion-induced reduction of fluorescence in the contact area and its simultaneous increase in the non-contact area follow a first order kinetic with about the same rate constant of 0.03 s^−1^, corresponding to a half time *t*_1/2_ = 23 s (Fig. 3c, left panel).

Although Ca^2+^ depletion by the addition of EGTA has been shown numerous times to dissolve cadherin-mediated cell–cell adhesion [1, 21, 38], no obvious changes were seen in the relative positions of the cells within the short time frame analyzed in this study (Supplement Videos 1 and 2). However, as is depicted in the right panel of Fig. 3c, cell membranes that are kept in tricellular junctions under physical stress, i.e., high curvature, by LI-YFP-mediated *trans*-interactions, separate readily in the center within less than 20 s after Ca^2+^ depletion, relieving thus the physical strain on the plasma membrane. After that, no major change was observed within the next 19 min (Supplement Video 2).

Strikingly, the FRET efficiency of LI-cadherin remained unchanged upon Ca^2+^ removal (Fig. 3d), giving compelling evidence that Ca^2+^ has no influence on *cis*-dimerization. Furthermore, LI-cadherin that had diffused out of the dissolved cell–cell contact sites still exhibited the same FRET efficiency, indicating that it diffuses in the cell membrane as a functional *cis*-dimer (Fig. 3d). Upon restoring 2 mM Ca^2+^ in the buffer, LI-YFP readily re-accumulates in the previously dissolved cell–cell contacts (data not shown).

### Lateral diffusion of LI-cadherin depends on Ca^2+^

**Fig. 4:**
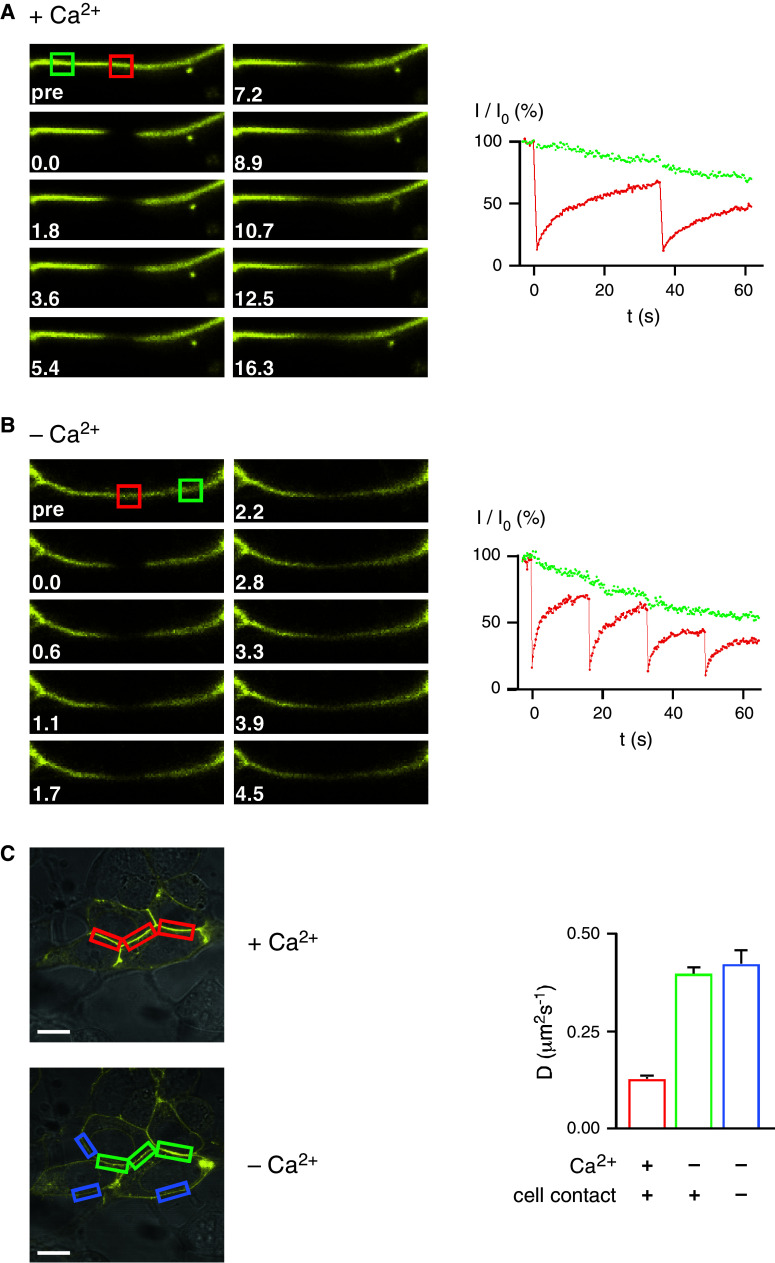
Lateral diffusion of LI-cadherin in the plasma membrane. **a** FRAP of LI-YFP expressed in HEK293 cells in the presence of Ca^2+^. *Left* time course of fluorescence image (frame width 12.9 μm) of a contact area between two transfected cells subsequent to photobleaching at *t* = 0. The bleached region is marked by a *red square*, an unbleached reference region by a *green square*. *Right* time-dependent graph of the FRAP experiment with region of interest inside (*red*) and outside (*green*) the bleaching area. Intensity data (*I*) are corrected for bleaching artefacts due to the data recording. **b** FRAP of LI-YFP expressed in HEK293 cells after EGTA induced Ca^2+^ removal. After the exchange of the standard medium by Ca^2+^-free medium and the addition of EGTA to a final concentration of 2 mM, FRAP was performed as described in **a**. Micrograph frame width 12.1 μm. **c** Diffusion coefficients of LI-YFP in the plasma membrane. *Left* confocal laser-scanning microscopy of LI-YFP-expressing HEK293 cells prior (*above*) and after Ca2^+^ removal and the addition of 2 mM EGTA (*below*). The *colored boxes* indicate typical regions of interest for FRAP experiments: cell–cell contact areas in the presence of Ca^2+^ (*red bar*), the same areas after Ca^2+^ depletion (*green bar*), and membrane areas not involved in cell–cell contacts (*blue bar*). *Scale bars* 10 μm. *Right* *bar diagram* displaying the results of the Ca^2+^-dependent FRAP experiments within the three different types of membrane areas as indicated by the same color code. Diffusion coefficients in means ± SE

The lateral mobility of LI-cadherin in the plasma membrane was quantified by FRAP. Unexpectedly, LI-cadherin (LI-YFP) fully redistributed after photobleaching in the cell–cell contact sites within a few minutes (Supplementary Video 1), exhibiting a diffusion coefficient of 0.124 ± 0.004 μm^2^/s (Fig. 4a, c; Supplementary Video 3). Upon EGTA-induced Ca^2+^ depletion, the diffusion coefficient rose almost threefold and was similar inside (0.395 ± 0.013 μm^2^/s) and outside (0.420 ± 0.033 μm^2^/s) the previous cell–cell contacts (Fig. 4b, c; Supplementary Video 4). This observation indicates that cell–cell contacts are readily lost upon Ca^2+^ depletion, although the opposing membranes usually do not separate as quickly. We conclude that LI-cadherin constitutively forms dimers that freely diffuse in the plasma membrane due to the lack of cytoplasmic interactions. In the presence of Ca^2+^ those functional dimers become quickly trapped in cell–cell contact sites and accumulate therein.

## Discussion

In our current study, we discovered that non-classical LI-cadherin *cis*-dimerizes constitutively on the surface of living cells. This result was obtained by FRET analysis, a powerful method to study molecular interactions within living cells [39]. In analogy to the successful FRET analysis of integrin *cis*-dimerization [40], we attached the required fluorophores, CFP and YFP, to the intracellular C terminus of LI-cadherin. We had previously shown that the short intracellular domain of LI-cadherin does not interact with cytoplasmic components and can even be deleted without altering the cell–cell adhesive function of the protein [20]. Although it is thus conceivable that the fluorescent protein tag will not interfere with LI-cadherin function, we generated stably transfected CHO cell lines expressing either LI-YFP or free YFP and verified their cellular morphology and cell adhesive properties in comparison to previously generated CHO clones expressing unmodifed LI-cadherin [21] and found no differences. Attaching fluorescent protein tags to the intracellular C terminus of cadherins thus appears to interfere much less with their function than inserting them in the extracellular domain, an approach that was recently successfully applied to study the *trans*-interactions of cadherins by FRET [41].

Whereas *cis*-dimerization has never been shown before for a non-classical cadherin like LI-cadherin, it has been assumed since a long time to exist in classical cadherins. On the one hand, the parallel interaction observed in the first crystal structure of a cadherin repeat (EC1 of N-cadherin) was interpreted as to stabilize a *cis* ‘strand dimer’ [6] and on the other hand N-terminal *cis*-interactions were clearly seen in electron micrographs of C-terminally pentamerized E-cadherin ectodomains [23]. In addition, *cis*-dimerization was also found for membrane integrated E-cadherin by cross-linking experiments [42]. However, a recent attempt to directly demonstrate the existence of *cis*-dimerization by single molecule FRET analysis of chemically fluorophore-labeled E-cadherin ectodomains failed [43]. This result was supported by a theoretical study based on the crystal structure of the complete C-cadherin ectodomain, which suggested that classical cadherins exist in the plasma membrane primarily as monomers and *cis*-dimerize only after engaging in *trans*-contacts [44].

In contrast to these findings, LI-cadherin *cis*-dimerizes constitutively on the cell surface as is revealed by the identical FRET efficiencies found for LI-CFP/LI-YFP within and outside cell–cell contacts. These data show that *cis*-dimerization is not secondary to the formation of *trans*-interactions and is also neither enhanced nor inhibited by *trans*-binding, as has been suggested before for classical cadherins [45]. Moreover, the substitution of Ca^2+^-containing buffer by Ca^2+^ free buffer and the subsequent addition of 2 mM EGTA also had no measurable influence on the FRET efficiencies. Since we had previously shown that *trans*-binding of LI-cadherin does not take place under those conditions [22], *cis*-dimerization of LI-cadherin is clearly independent of both Ca^2+^ and *trans* interactions. In contrast to classical E- and N-cadherin, LI-cadherin does not contain a tryptophan near the N terminus of its first extracellular repeat EC1. Thus, we have to rule out that strand-swapping is required for *cis*-dimerization of LI-cadherin. This conclusion is in line with the recent report that W2 is involved in stabilizing *trans*-interactions rather than *cis*-dimerization [27]. The role of Ca^2+^ for *cis*-dimerization of classical cadherins has not been unequivocally resolved. Some studies revealed a Ca^2+^-independent *cis*-dimerization [42, 46] whereas other experiments showed that Ca^2+^ is essential for *cis*-dimerization [24, 47]. However, since *cis*-dimerization of non-classical LI-cadherin may involve different mechanisms, our results cannot resolve this dispute.

In the presence of Ca^2+^, LI-cadherin highly concentrates in the cell–cell contact regions of adjacent cells. Upon Ca^2+^ depletion, it leaves within a few minutes (*t*_1/2_ less than 30 s) those contact sites and redistributes—still as a *cis*-dimer—evenly on the entire cell surface. This finding indicates that LI-cadherin is kept in the cell–cell contact sites solely by its *trans*-interactions that are immediately dissolved upon decreasing the Ca^2+^ concentration below 0.7 mM [22]. In order to quantify the mobility of LI-cadherin, we performed FRAP experiments and determined a diffusion coefficient of 0.420 ± 0.033 μm^2^/s for the freely diffusing LI-cadherin dimer outside cell–cell contacts. This value falls into the range one would expect for the unrestricted diffusion of a plasma membrane-integrated protein of this size [48]. However, for E-cadherin, which firmly interacts with β-catenin, a much slower diffusion coefficient has been revealed on the cell surface by single particle tracking (0.002–0.006 μm^2^/s) [49, 50], FRAP using fluorescent monoclonal antibodies (0.003 μm^2^/s)[49], FRAP using a cytoplasmic GFP fusion protein (0.036 μm^2^/s) [8], and single molecule FRAP with an analogous construct (0.028 μm^2^/s) [51]. In addition, a large fraction, reaching 90 % in mature E-cadherin plaques, was found to be immobile [8] or to diffuse 40 times slower (0.0007 μm^2^/s) [51]. It has been assumed that the immobile fraction of E-cadherin is associated with the cytosceleton [8].

It was thus surprising to find that LI-cadherin engaged in *trans*-binding within cell–cell contact sites still exhibits high diffusion coefficient of 0.124 ± 0.004 μm^2^/s, just three times smaller than that outside of the contacts, but more than 150 times higher than was reported for classical E-cadherin [51]. Therefore, LI-cadherin is still highly mobile within cell–cell contact sites. This observation can be attributed to two properties of LI-cadherin: On the one hand, it does not firmly interact with any cytoplasmic components [20], and on the other hand, its *trans*-binding exhibits a short half time of *t*_0_ = 1.41 s [22]. *Trans*-binding of LI-cadherin is thus highly dynamic and the interactions of individual LI-cadherin molecules are assumed to be constantly broken and formed. This mechanism may enable LI-cadherin-expressing cells, i.e., enterocytes, to adopt quickly to sudden changes in physical stress, as is typical for the intestinal epithelium that is experiencing sheer stress by abrupt flows of liquid and gas, lateral tension by different intestinal filling pressures, and changes in osmotic pressure upon intake of large amounts of water with low osmolarity [52, 53].

### Electronic supplementary material

Below is the link to the electronic supplementary material.

Supplementary material 1 (MPG 1502 kb)

Supplementary material 2 (MPG 1226 kb)

Supplementary material 3 (MPG 3026 kb)

Supplementary material 4 (MPG 5612 kb)

## Abbreviations

AFM: Atomic force microscopy
CFP: Cyan fluorescent protein
CHO: Chinese hamster ovary (cells)
EC1: Extracellular cadherin repeat 1
EC12: Extracellular cadherin repeats 1 and 2
FRAP: Fluorescence redistribution after photobleaching
FRET: Fluorescence resonance energy transfer
HBS: HEPES-buffered saline
HEK293: Human embryonic kidney (cells)
LI-CFP: LI-cadherin-CFP fusion protein
LI-YFP: LI-cadherin-YFP fusion protein
LSM: Laser scanning microscopy
SE: Standard error (of the mean)
τ_0_: Lifetime (of a molecular interaction)
W2: Tryptophan 2
YFP: Yellow fluorescent protein
